# A Case, in Which a Collection of Fluid and Coagulable Matter, to the Amount of Several Pints, Took Place between the Membranes of the Brain

**Published:** 1834-07-01

**Authors:** John Howship

**Affiliations:** 21, Saville Row


*To the Editor of the Medical Quarterly Review.*

SIR: Should the following short account of a case, which appeared during life to be only a case of hydrocephalus, be deemed interesting, it is very much at your service.

I am, sir, your obedient servant,

JOHN HOWSHIP.

21, *Saville Row; May* 20, 1834.

*A Case, in which a Collection of Fluid and Coagulable Matter, to the amount of several Pints, took place between the Membranes of the Brain.*

July 12, 1826. In presence of Dr. James, Dr. Elliotson, and Mr. Holberton, I examined the head and body of James Gulliver, aged four years, who had died in St. George's Infirmary, with hydrocephalus, as was supposed. The little boy had enjoyed good health till about six months old, when enlargement of the head commenced. I saw him a few months before he died, and, with the exception of the enlarged state of the head, his health then appeared excellent: he was lively, and particularly intelligent, and his mother said that the child's intellects were exceedingly bright. He had sunk, and died, from irritable and relaxed bowels.

The head, at the time of his death, measured twenty-eight inches in circumference.

On removing the calvarium, although the opening of the fontanelle was not closed, the skull was in most parts as thick, though not so hard, as it is usually found at the age of twenty years. The dura mater was most firmly attached to the inside of the cranium. In fact, the difficulty in separating them induced me carefully to cut through the right side of the dura mater, in order to detach the superior portion of the skull, when we were surprised by the flowing out of a quantity of turbid bloody serum. The calvarium, with the dura mater, removed, we after some time ascertained what appeared to be the true state of the case.

The dura mater lining the superior part of the skull was much thickened; externally, at certain points, discoloured, inflamed, or of a lurid tinge; and internally covered, on every part of its surface, with a layer of coagulable lymph; and within that, on most parts, was a quantity of a loose albuminous secretion, of a brick red colour, exactly similar to that of the serous fluid with which the large space within the dura mater was filled. The grumous appearance of the albuminous deposit was apparently derived from the colouring matter of the blood.

The convex floor of this extensive cavity, which occupied the whole of the superior and lateral parts of the skull, was also thickly covered with this loose, grumous deposit, of which I collected and laid aside at least one pound. On dividing this convex floor, it proved to be the arachnoid membrane and pia mater, beneath which lay the brain, of healthy structure, though disturbed, from the two lateral ventricles being thrown into one cavity by a collection of serum. The ventricles contained as much as eight or ten ounces of transparent fluid, slightly tinged with the bright crimson colour of arterial blood; affording a very striking contrast to the dirty brick-coloured deposit external to the brain, although the colour in both fluids must have been derived from the same source and principle.

The entire quantity of fluid collected from within the head measured four and a half pints. It was curious that there was not the least trace of purulent matter found in the course of the examination.

The general opinion of the gentlemen present appeared to be, that the whole of the disease had originated in the arachnoid membrane, although in health it possesses no red vessels, and although, even in the present case, the expansion of the arachnoid lining the cavities of the ventricles was free from any trace of disease. My own impression, however, rather induced me to refer these exceedingly unusual appearances to some inflammatory action in the dura mater, seeing that the whole of the internal surface of this membrane, and this membrane only, was covered by a layer of effused fibrine, while its external surface was discoloured, from augmented vascularity; and seeing also that even the increased deposit of bone in the cranium was such as might be expected to attend not only a state of considerable, but of long-continued vascular excitement in that membrane, the blood-vessels of which may be regarded as the more immediate agents in the formation of the bones of the skull.

I have frequently seen copious effusions of flocculent or of laminated fibrine from the inner surface of the dura mater, and have also occasionally observed a modified effusion of fibrinous matter on the outer surface of the tunica arachnoides; but the violence of the action in the one case, contrasted with the partial and sparing result in the other, only tends to strengthen the probability that, in the present instance, the most remarkable appearances were induced by some increased action in the vessels of the dura mater.

I carefully removed and injected the alimentary canal; but, on subsequently opening and examining the internal surface, I only detected four or five small, sloughy, ulcerated spots, the size of coriander-seeds, in the transverse arch of the colon. The other viscera were healthy.

